# Cognitive Behavior Therapy as Augmentation for Sertraline in Treating Patients with Persistent Postural-Perceptual Dizziness

**DOI:** 10.1155/2018/8518631

**Published:** 2018-03-07

**Authors:** Yi-Chuan Yu, Hui Xue, Ying-xin Zhang, Jiying Zhou

**Affiliations:** ^1^Department of Emergency, Yongchuan Hospital of Chongqing Medical University, Chongqing, China; ^2^Department of Neurology, Baotou Central Hospital, Inner Mongolia, China; ^3^Qinggang Senior Care Center, The First Affiliated Hospital of Chongqing Medical University, Chongqing, China; ^4^Department of Neurology, The First Affiliated Hospital of Chongqing Medical University, Chongqing, China

## Abstract

**Background:**

Persistent postural-perceptual dizziness (PPPD) is a common vestibular disorder. This study was conducted to assess whether the addition of cognitive behavior therapy (CBT) could significantly improve the efficacy and acceptability of sertraline in treating PPPD.

**Methods:**

PPPD patients were recruited and randomly assigned to control and experiment groups. Patients in both groups received sertraline 50–200 mg/day, and only patients in the experiment group received CBT (twice a week, one hour per time). The treatment was continued for eight weeks. At baseline, week 2, week 4, and week 8, the 25-item Dizziness Handicap Inventory (DHI), Hamilton Anxiety Rating Scale (HARS), and Hamilton Depression Rating Scale (HDRS) were used to assess the self-perceived handicapping effects caused by PPPD, anxiety, and depressive symptoms, respectively. The dose of sertraline used and the adverse events in both groups were recorded and analyzed.

**Results:**

In total, 91 PPPD patients were randomly assigned to the control group (*n* = 45) and experiment group (*n* = 46). After eight weeks of treatment, the average DHI scores, HDRS scores, and HARS scores were significantly decreased in both groups. But compared to the control group, the experiment group had significantly lower average DHI score, HDRS score, and HARS score at weeks 4 and 8. Moreover, the dose of sertraline used in the experiment group was significantly lower than that in the control group, and adverse events occurred more frequently in the control group than in the experiment group (48.9% versus 26.1%, *p* = 0.025).

**Conclusion:**

These results demonstrated that the addition of CBT could significantly improve the efficacy and acceptability of sertraline in treating PPPD and reduce the dose of sertraline used.

## 1. Introduction

Persistent postural-perceptual dizziness (PPPD) is a syndrome of subjective imbalance or chronic nonvertiginous dizziness accompanied by hypersensitivity to motion stimuli and poor tolerance for complex visual stimuli or precision visual tasks, but without identifiable vestibular impairments [1, 2]. The continual subjective imbalance or nonvertiginous dizziness persisting for three months or longer is the core physical symptom of this disease [3]. Patients with PPPD often describe the dizziness as swaying or rocking when standing or sitting. This disease could seriously affect the quality of life of patients. Although PPPD has been widely accepted by American clinicians, it is currently not well understood by domestic clinicians [4]. Actually, PPPD is very common in clinical practice, which accounts for about 10% of outpatients with dizziness; among these PPPD patients, the number of female patients is twice that of male patients [5].

Up till now, some treatment modalities, such as medication [6–9], have been established for PPPD. Selective serotonin reuptake inhibitors (SSRIs), such as sertraline, paroxetine, and fluoxetine, have been found to reduce the symptoms of PPPD [7–9]. In fact, antidepressant medications, predominantly SSRIs, are currently the most common treatment for PPPD. However, not all patients could benefit from treatment with SSRIs. For instance, a previous study reported that about 36% of patients treated with SSRIs did not experience any benefit or could not tolerate the medication because of adverse events [6]. Meanwhile, vestibular rehabilitation (VR), a nonpharmacological therapy, has also been shown to improve the outcomes in patients with dizziness [10, 11]. It could facilitate central nervous system compensation for vestibular impairments after specific exercises [12]. But the mechanisms that underlie the symptom reduction in patients without vestibular impairments (whose symptoms are psychophysiological) after VR are unclear.

In recent years, many researchers have focused on the efficacy of cognitive behavior therapy (CBT) in treating patients with dizziness [13, 14]. Holmberg et al. found that the CBT had a limited long-term effect on phobic postural vertigo [13]. Edelman et al. found that the CBT could produce significant improvements in dizziness-related symptoms among patients with PPPD [14]. Generally speaking, CBT alone might be effective in treating less severe forms of mental disorders, but it was often recommended in combination with other therapy methods in treating health problems in clinical practice. For instance, CBT is often recommended in combination with medications, such as serotonin-norepinephrine reuptake inhibitors (SNRIs) and SSRIs, for treating neuropsychiatric disorders. Previous studies reported that the addition of CBT could improve the efficacy of these medications in treating depression and anxiety [15–17]. Considering the close relationship between psychological symptoms and PPPD, we hypothesized that the CBT could significantly improve the efficacy and acceptability of sertraline in treating patients with PPPD, and we conducted this randomized controlled trial to verify this hypothesis.

## 2. Methods

### 2.1. Subjects and Procedures

This study was reviewed and approved by the Ethical Committee of Chongqing Medical University (Chongqing, China) and is in accordance with the Declaration of Helsinki. Patients who met the following inclusion criteria were recruited: (i) a diagnosis of PPPD (based on unexplained symptoms including subjective imbalance, dizziness/lightheadedness, and exacerbation in busy public places); (ii) age between 18 and 60 years.; (iii) not using any psychotropic medications or antidizziness drugs two weeks prior to study entry; (vi) >=7 Hamilton Anxiety Rating Scale (HARS) score [18] and >=17 Hamilton Depression Rating Scale (HDRS) (17-item) score [19]; and (v) providing written informed consent. Meanwhile, patients who met the following exclusion criteria were excluded: (i) having an organic brain disorder, severe physical illness, or other major mental illnesses; (ii) alcohol or substance abuse or dependence; (iii) having suicidal ideation or significant cognitive deficit; and (iv) pregnant or lactating women. The first participant was randomized in February 2014 and the last clinic visit occurred in March 2017.

### 2.2. Treatment Methods

The recruited patients were randomly assigned to the control group or the experiment group. Randomization was conducted using a random number sequence. Patients in both groups received sertraline in the morning, which started at 50 mg/day and could increase by 50 mg increments to a maximum of 200 mg/day within four weeks, if needed. At the end of the trial, in order to avoid the potential unpleasant adverse reactions, the dose of sertraline was gradually reduced rather than abruptly discontinuing the treatment. Meanwhile, patients in the experiment group received CBT, twice a week, one hour per time. The main components of CBT included (i) earning the trust of patients; (ii) encouraging the patients to communicate with others; (iii) making patients expose and check the social factors that cause the PPPD, such as family, work, and social intercourse; and (iv) making patients have a correct understanding of the occurrence, development, and treatment of PPPD. The psychologist performed a psychological intervention by releasing some positive signals to patients and changed their formed pathological defense mechanisms by adjusting their cognition. The treatment was continued for eight weeks.

### 2.3. Measurement Index

The HDRS and HARS were used to assess the depressive and anxiety symptoms of patients, respectively, at baseline, week 2, week 4, and week 8. Higher HDRS and HARS scores indicated more severe depression and anxiety, respectively. The 25-item Dizziness Handicap Inventory (DHI) was used to assess the self-perceived handicapping effects caused by vestibular system disease at baseline, week 2, week 4, and week 8 [20]. Higher DHI scores indicated the severe impact of dizziness on everyday life. Meanwhile, in order to assess whether or not the addition of CBT could reduce the dose of sertraline, the dose of sertraline used in both groups was calculated and analyzed. The adverse events were also recorded to assess the safety of the two different treatment modalities.

### 2.4. Statistical Analysis

Mean and standard deviation were used to express the data that was characterized by a normal distribution. Student's *t*-test and Chi-squared test were conducted when appropriate. Repeated measures analysis of variance (ANOVA) was conducted to explore the group differences on HDRS, HARS, and DHI scores at four time points. All analyses were two-tailed and conducted using SPSS 19.0 (SPSS Inc., Chicago, IL, USA), and the level of significance was set at *p* < 0.05.

## 3. Results

### 3.1. Demographic Profile

In total, 109 PPPD patients met the inclusion/exclusion criteria. Among these patients, there were eight patients refusing to participate due to personal or family reasons, six patients refusing to be randomly assigned, and four patients failing to complete the interview. These patients were excluded from this study. Finally, 91 PPPD patients were randomly assigned to the control group (*n* = 45) and experiment group (*n* = 46). The baseline data of patients, such as age, sex, body mass index (BMI), and duration of dizziness, were similar between the two groups. The detailed information is described in Table 1.

### 3.2. Dizziness Handicap Inventory

**Figure 1:**
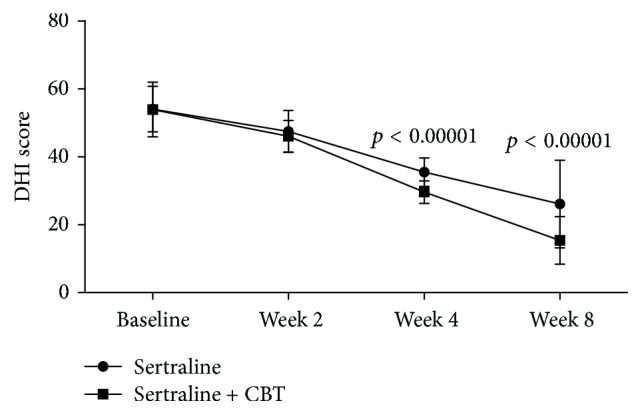


The average DHI scores decreased over time in both the control and the experiment groups (Figure 1). The repeated measures ANOVA showed a significant effect of time (*p* < 0.00001), which indicated that both sertraline as monotherapy and sertraline + CBT could significantly reduce chronic subjective dizziness. Meanwhile, the repeated measures ANOVA also showed a significant effect of group × time interaction (*p* < 0.00001), which indicated that the reductions were significantly different between the control and experiment groups. As shown in Figure 1, the average DHI scores were similar between the two groups at baseline and week 2, but at week 4 and week 8, the average DHI scores in the experiment group were significantly lower compared to the control group (*p* < 0.00001, *p* < 0.00001). These results showed that sertraline + CBT could be more effective in reducing chronic subjective dizziness compared to sertraline as monotherapy.

### 3.3. Depressive Symptoms

**Figure 2:**
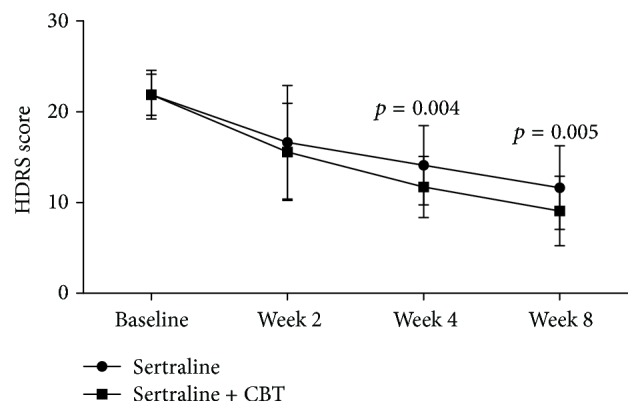


The average HDRS scores decreased over time in both the control and the experiment groups (Figure 2). The repeated measures ANOVA showed a significant effect of time (*p* < 0.00001), which indicated that both sertraline as monotherapy and sertraline + CBT could produce significant reductions in depressive symptoms. Meanwhile, the repeated measures ANOVA also showed a significant effect of group × time interaction (*p* = 0.029), which indicated that the reductions were significantly different between the control and experiment groups. As shown in Figure 2, the average HDRS scores were similar between the two groups at baseline and week 2, but at week 4 and week 8, the average HDRS scores in the experiment group were significantly lower compared to the control group (*p* = 0.004, *p* = 0.005). These results showed that sertraline + CBT could produce more reductions in depressive symptoms.

### 3.4. Anxiety Symptoms

**Figure 3:**
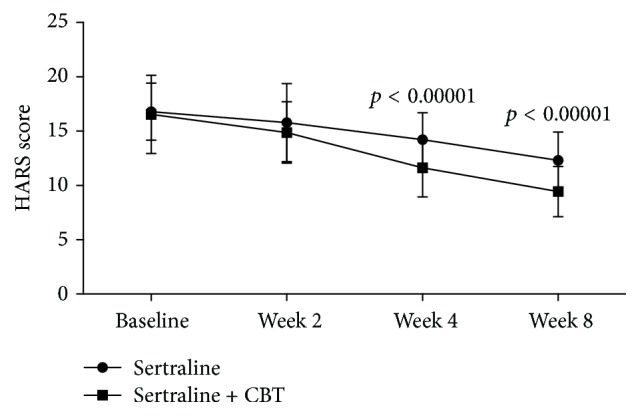


The average HARS scores decreased over time in both the control and the experiment groups (Figure 2). The repeated measures ANOVA showed a significant effect of time (*p* < 0.00001), which indicated that both sertraline as monotherapy and sertraline + CBT could produce significant reductions in anxiety symptoms. Meanwhile, the repeated measures ANOVA also showed a significant effect of group × time interaction (*p* = 0.002), which indicated that the reductions were significantly different between the control and experiment groups. As shown in Figure 3, the average HARS scores were similar between the two groups at baseline and week 2, but at week 4 and week 8, the average HARS scores in the experiment group were significantly lower compared to the control group (*p* < 0.00001, *p* < 0.00001). These results showed that sertraline + CBT could produce more reductions in anxiety symptoms.

### 3.5. Sertraline Dose

**Figure 4:**
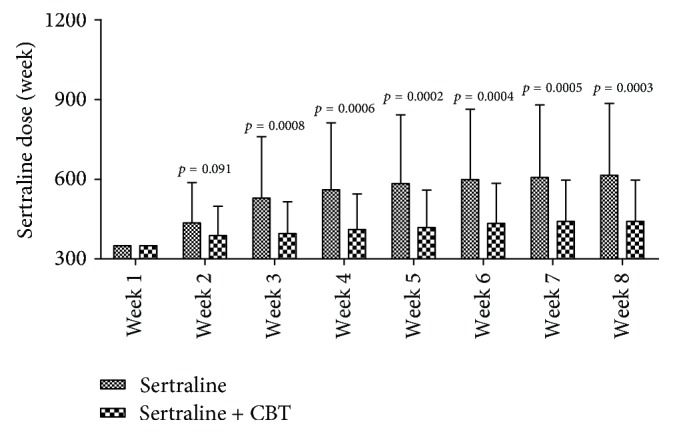


As shown in Figure 4, the average dose of sertraline used in one week increased from 350 mg/week to 614.44 mg/week in the control group and from 350 mg/week to 441.30 mg/week in the experiment group. At week 1, patients in both groups received sertraline 50 mg/day. At week 2, the difference in the average dose of sertraline used between the two groups was not statistically significant (*p* = 0.091), although the average dose of sertraline used in the experiment group was lower. But compared to the control group, the experiment group needed significantly lower average dose of sertraline at week 3 (*p* = 0.0008), week 4 (*p* = 0.0006), week 5 (*p* = 0.0002), week 6 (*p* = 0.0004), week 7 (*p* = 0.0005), and week 8 (*p* = 0.0003). These results indicated that the addition of CBT could effectively reduce the dose of sertraline used.

### 3.6. Adverse Events

One patient in the control group developed acute suicidal intent at week 7; however, there were no suicides. The following adverse events in the control group were reported during the whole treatment period: nausea (*n* = 4), vomiting (*n* = 3), indigestion (*n* = 2), sweating (*n* = 2), diarrhea (*n* = 4), insomnia (*n* = 2), somnolence (*n* = 2), constipation (*n* = 1), irritability (*n* = 2), headache (*n* = 3), dry mouth (*n* = 3), and sexual side effects (*n* = 2). The following adverse events in the experiment group were reported during the whole treatment period: nausea (*n* = 2), vomiting (*n* = 2), indigestion (*n* = 1), diarrhea (*n* = 2), somnolence (*n* = 1), constipation (*n* = 1), headache (*n* = 1), dry mouth (*n* = 2), and sexual side effects (*n* = 2). The proportion with any adverse events was significantly higher in the control group (48.9%) compared with the experiment group (26.1%) (*p* = 0.025). These results indicated that sertraline + CBT could be safer and more acceptable than sertraline as monotherapy.

## 4. Discussion

This study was conducted to investigate whether the addition of CBT could significantly improve the efficacy and acceptability of sertraline in treating PPPD. After eight weeks of treatment, both sertraline as monotherapy and sertraline + CBT could significantly reduce the average DHI scores, HDRS scores, and HARS scores, which indicated that both treatment methods were effective in treating PPPD. But compared to sertraline as monotherapy, sertraline + CBT could yield significantly lower average DHI score, HDRS score, and HARS score. Meanwhile, both the average dose of sertraline used in each week (from week 3 to week 8) and the incidence of adverse events in the experiment group were significantly lower than those of the control group. These results demonstrated that the addition of CBT could significantly improve the efficacy and acceptability of sertraline in treating PPPD, and this method needed a significantly lower dose of sertraline.

Sertraline was chosen because it had the most favorable balance between acceptability, benefits, and acquisition cost in treating depression among the twelve new-generation antidepressants [21]. And there was a close relationship between PPPD and psychiatric or neurologic illnesses [4]. In addition, sertraline was primarily metabolized by the liver. N-desmethylsertraline, as its active metabolite, was further metabolized into an inactive form prior to renal excretion [22]. Moreover, a randomized trial found that sertraline was safe in patients with cardiovascular disease [23]. Although a previous study found a significantly higher incidence of gastrointestinal adverse events in patients receiving sertraline versus placebo, those treated with sertraline did not experience serious adverse events or bleeding [24].

CBT was a psychosocial intervention that had been the most widely used practice for improving mental health [25]. It focused on developing the personal coping strategies, which mainly target changing unhelpful patterns in behaviors, cognition, and emotional regulation [26]. CBT was originally designed to treat depression, which is a disease caused by many factors [27, 28]. But nowadays, it is used to treat a number of mental health conditions. Due to the difference in duration and content, previous studies reported some different results when using CBT to treat PPPD [14, 29]. Edelman et al. found that CBT alone could not produce significant changes in psychologic outcomes (anxiety, depression, and stress) [14]. But Tian-yi et al. reported that CBT alone could significantly reduce the depression and anxiety symptoms [29]. Here, we found that the combination of CBT and sertraline was more effective in reducing chronic subjective dizziness and depressive and anxiety symptoms. These findings might suggest that it was a good choice to combine application of CBT and other therapy methods in treating PPPD.

There were a number of limitations in our study. The sample size was relatively small, which might have limited available power and ability to identify the more subtle changes, although significant improvements were found on the three measurement scales. Another limitation was that the treatment was only continued for eight weeks. Thus, the long-term effects of sertraline as monotherapy versus sertraline + CBT were not assessed here. Future studies are needed to measure the outcomes over longer periods. A further limitation was that the CBT was not blinded to the patients, which might cause “unreal” better results in the experiment group. Finally, there was no placebo-control group in this study. Then, additional studies are still required to compare sertraline + CBT with placebo-control condition. Moreover, future studies should compare the relative effectiveness of other therapy methods, such as VR, CBT, other SSRIs, or combination treatments, which might help to find the optimal therapy methods for particular patients.

In conclusion, the outcomes of our study demonstrated that the addition of CBT could significantly improve the efficacy and acceptability of sertraline in treating PPPD. Moreover, the addition of CBT could significantly reduce the dose of sertraline, which could generate substantial cost savings to the individual and community. Despite being limited by the relatively small sample size, our findings would add to the body of knowledge on the intervention methods for PPPD and could contribute to the development of more effective therapy methods over time.

## Figures and Tables

**Table 1:** Demographic profile of the recruited CSD patients.

Variables	Control group	Experiment group	*p* value
*n*	45	46	-
Age (year)	42.16 (9.62)	42.67 (9.75)	0.79
Female/male	32/13	30/16	0.55
Education (year)	7.13 (0.2.99)	7.04 (3.01)	0.88
BMI (kg/m^2^)	23.14 (3.28)	22.79 (3.56)	0.63
Duration of CSD (year)	1.77 (0.0.78)	1.82 (0.73)	0.75
Smoking (Y/N)	12/33	18/28	0.21
Single household (Y/N)	10/35	13/33	0.51
DHI scores	54.02 (6.70)	53.91 (8.05)	0.94
HDRS scores	23.77 (2.83)	23.76 (2.93)	0.97
HARS scores	18.77 (2.69)	18.54 (3.60)	0.72
